# Construction and Immunological Evaluation of a Bicomponent Zika mRNA Vaccine

**DOI:** 10.3390/vaccines14100897

**Published:** 2026-10-13

**Authors:** Qi Su, Tingyi Che, Shuai Shang, Xinzhu Wang, Xiaoqin Ge, Fang Cai, Hongyu Wang, Hongshan Xu, Qunying Mao, Xiaohong Wu, Xinyu Liu

**Affiliations:** 1 Changchun Bioproducts Research Co., Ltd., Changchun 130103, China; rivaille_sq@foxmail.com (Q.S.); euemsh@foxmail.com (T.C.); shangshuai2026@163.com (S.S.); wupcp73@163.com (X.W.); 2 National Institutes for Drug Control, Beijing 102629, China; wanghongyu0600@163.com (H.W.); xuhongshan@nifdc.org.cn (H.X.);; 3 SINOVAC BIOTECH CO., LTD., Beijing 102629, China; gexq@sinovac.com (X.G.); caif@sinovac.com (F.C.)

**Keywords:** bicomponent vaccine, ZIKV, E Domain III, NS1, mRNA

## Abstract

Background: There is evidence for the protective effects of antibodies targeting non-structural protein 1 (NS1) of Zika virus (ZIKV); however, effective vaccines against ZIKV remain lacking. Methods: In this study, lipid nanoparticle (LNP)-encapsulated mRNA vaccines were designed based on the envelope domain III (EDIII) and NS1 sequences of the ZIKV FSS13025 strain, while protective efficacy was evaluated in a murine model using a ZIKV isolate obtained from Zhejiang Provincial Center for Disease Control and Prevention (Zhejiang CDC). Results: The vaccines were evaluated for key quality attributes—including particle size, polydispersity index (PDI), and encapsulation efficiency—as well as immunogenicity and protective efficacy. Both monovalent vaccine components exhibited uniform particle sizes (PDI < 0.2) and high encapsulation efficiencies (≥80%) and induced high levels of antigen-specific antibodies in mice in a dose-dependent manner. The EDIII mRNA vaccine elicited moderate ZIKV-neutralizing antibody titers as measured by PRNT_50_, whereas the NS1 vaccine did not induce detectable neutralizing antibodies. Both bicomponent (EDIII + NS1) mRNA vaccines induced a Th1-biased cellular immune response in mouse splenocytes, with significantly greater IFN-γ production compared with either monovalent vaccine. In viral challenge experiments using 1 × 10^5^ PFU ZIKV (Zhejiang CDC isolate, intraperitoneal), the bicomponent vaccine provided complete protection, with 100% survival across all dose groups. In contrast, the EDIII and NS1 monovalent vaccines showed partial protection: mortality was observed in the 20 μg dose groups and exceeded 50% in the 10 μg dose groups. Conclusions: These results demonstrate that the bicomponent EDIII + NS1 mRNA vaccine combination confers superior protective efficacy compared with either component alone, consistent with a synergistic effect between humoral and cellular immune responses. This study provides a novel strategy for next-generation Zika vaccine development.

## 1. Introduction

The Zika virus (ZIKV), a single-stranded positive-sense RNA virus belonging to the family Flaviviridae, exhibits diverse transmission routes, including mosquito bites (the primary route) as well as vertical (mother-to-child) and sexual transmission [1,2]. In adults, infection typically manifests as self-limiting fever but may trigger severe conditions, such as Guillain–Barré syndrome. It poses particularly severe risks to fetuses, potentially causing placental insufficiency, congenital malformations (such as microcephaly), preterm birth, or miscarriage [3]. Following a major outbreak in South America in 2016, ZIKV garnered widespread attention; however, no licensed vaccine is currently available [4].

The structure of mature ZIKV particles exhibits distinct hierarchical features. The core contains an approximately 11 kb positive-sense single-stranded RNA genome, encapsidated to form the nucleocapsid and surrounded externally by a lipid membrane (viral envelope) [5]. On the surface of the lipid membrane, 180 complete envelope glycoproteins (E proteins) are uniformly distributed; these E proteins do not exist in isolation but assemble as 90 homodimers, arranged in a herringbone symmetry pattern, ultimately fully covering the outer surface of the viral particle and forming the critical structural basis for virus–host cell interactions [6,7].

The ZIKV E protein is the primary target of neutralizing antibodies [8]. The mature ZIKV E protein consists of three domains (EDI, EDII, and EDIII) [9]. Among these, the EDIII domain exhibits the highest antigenic specificity, and antibodies targeting this domain (nAbs) are less likely to cross-react with other FVs. Therefore, a vaccine derived from the EDIII domain may help reduce the risk of ADE associated with DENV, making EDIII an important key target for Zika vaccine candidate design [10,11,12,13].

ZIKV encodes seven nonstructural proteins (NS1, NS2A, NS2B, NS3, NS4A, NS4B, and NS5) [7]. NS1, a secreted protein, promotes ZIKV invasion of host cells. This protein is closely associated with ZIKV-induced neonatal microcephaly; it modulates glycosaminoglycans on trophoblasts and chorionic villi, leading to placental dysfunction and increased human placental permeability [14,15,16]. Studies have shown that vaccines designed solely with NS1 as the antigen can induce immunoprotective effects in AG129 mice [17]. Furthermore, the NS1 protein does not induce an ADE effect [13]. mRNA vaccines, benefiting from their flexible design, strong immunogenicity, and high safety, have become a major direction in Zika vaccine development [7,18,19,20,21]. This study employs mRNA vaccine technology to design and prepare a bicomponent (EDIII + NS1 protein) vaccine targeting ZIKV to reduce the ADE effect on DENV. The vaccine was evaluated in terms of humoral immunity, cellular immunity, and viral challenge protection against lethal challenge. The newly developed bicomponent mRNA vaccine showed robust immunogenicity, providing a basis for addressing the risk of ZIKV expansion [13,22].

## 2. Materials and Methods

### 2.1. mRNA Vaccine Preparation

The bicomponent mRNA vaccine encodes the EDIII domain and NS1 protein of the Zika virus (FSS13025 GenBank: JN860885). To enhance antigen expression and release, the native signal peptide of human tissue-type plasminogen activator (tPA) (selected from the albumin signal peptide) was incorporated at the 5′ end of the antigen proteins. The 5′ UTR (extracted from human α-globin RNA) contains a ribosome recognition sequence and works together with the 3′ UTR (extracted from 12s rRNA) to initiate mRNA translation and handle mRNA subcellular localization, while the polyadenylation (including 30 “A”, 10 any nucleotide + 70 “A”) at the 3′ end mainly keeps the mRNA stable. mRNA was synthesized in vitro using linearized DNA plasmids as templates. A cap analog was added to the mRNA to form a Cap-1 structure. This structure protects the mRNA from being degraded by nucleases by binding to translation initiation factors, while also helping to start translation and avoid being recognized by the natural immune system. DNase I was added to the reaction system to eliminate DNA templates and terminate the reaction.

All expression elements were cloned into a commercial vector plasmid. Following plasmid linearization, the mRNA solution was synthesized via in vitro transcription (IVT). Subsequently, the mRNA solution was mixed with lipid nanoparticle (LNP) components at a constant flow rate using INano^TM^ L (Micro & Nano, Shanghai, China) to generate mRNA-LNP formulations. The encapsulation efficiency was preliminarily evaluated by measuring particle size distribution and encapsulation rate.

### 2.2. Animals and Cells

Vero and HEK 293T cells were purchased from the American Type Culture Collection (ATCC) and maintained at the National Institutes for Drug Control (NIDC), China. Cells were cultured in DMEM (Dulbecco’s Modified Eagle Medium) supplemented with 10% fetal bovine serum and 1% antibiotics at 37 °C with 5% CO_2_. Beijing Sinovac Biotech Co., Ltd. (Beijing, China) provided the Zika virus strain used in the PRNT and challenge experiments; the virus was isolated in Zhejiang, China, in 2016.

The animal experimental protocol was approved by the NIDC Institutional Animal Care and Use Committee and conducted in accordance with its guidelines. Six-week-old specific pathogen-free female BALB/c and AG129 mice were provided and housed in the Institute for Laboratory Animal Research, NIDC, China. BALB/c mice were randomly assigned to EDIII, NS1, EDIII + NS1 vaccine, or placebo groups (PBS) at different doses (5, 10, and 20 μg) (*N* = 5 mice/group); the bicomponent vaccine was administered according to (2.5:2.5, 5:5, 10:10 µg) doses. BALB/c and AG129 mice were immunized via intramuscular injection into the tibialis anterior muscle with a volume of 50 μL per dose. Primary immunization was administered on day 0, with a booster on day 21 post-primary immunization. Serum was collected 35 days after primary immunization. Mice were subsequently euthanized to harvest splenocytes for downstream analyses. AG129 mice were randomly assigned to EDIII, NS1, EDIII + NS1 vaccine, or placebo groups at different doses (10 and 20 μg), boosted on day 21 post-primary immunization, with serum collected on day 35 and intraperitoneal viral challenge on day 42 (under intraperitoneal challenge with 10^5^ PFU ZIKV). To minimize distress in mice during blood collection, we anesthetized them via inhalation of isoflurane (cat#: R510-22-10, RWD) with an induction concentration of 3% and a maintenance concentration of 1%. Mouse body weights were recorded before the challenge and every 3 days post-challenge until the placebo group reached the humane endpoint (moribund or ≥20% body weight loss from baseline). Mice were euthanized by exposure to a high concentration of carbon dioxide gas.

### 2.3. In Vitro Biological Activity Assay

In summary, following the manufacturer’s instructions, Lipomaster 2000 Transfection Reagent (cat#: TL201-01, Vazyme, Nanjing, China) was used to transfect HEK293T cells (24-well plate: 2 μg, 1 μg, and 0 μg per well). To test for the expression of EDIII or NS1, cell lysates and culture medium were collected 48 h after transfection and analyzed using an enzyme-linked immunosorbent assay (ELISA).

### 2.4. Analysis of Cellular Immune Responses Using Enzyme-Linked Immunospot (ELISpot) Assays

Mice were euthanized and immersed in 75% ethanol for 1 min, after which spleen tissues were harvested into 10 cm cell culture dishes. Four to five milliliters of mouse lymphocyte separation medium (cat#: 7211011, Dakewe, Shenzhen, China) were added, and spleen tissues were ground using a 2 mL syringe plunger, followed by filtration through a 40 μm filter. The splenocyte suspension was immediately transferred to 15 mL centrifuge tubes, supplemented with 1 mL of RPMI-1640 medium (cat#: 42401042, Gibco, Waltham, MA, USA), and centrifuged at 800× *g* for 30 min at 4 °C. The solution in the centrifuge tube separated into four layers from top to bottom: RPMI-1640 medium, lymphocytes, a separation medium layer, and a layer containing erythrocytes and cell debris. After aspiration, the lymphocyte layer was added to 10 mL of the RPMI-1640 medium and centrifuged at 250× *g* for 10 min at 4 °C to harvest lymphocytes, which were then resuspended in serum-free medium. Mouse IFN-γ ELISpot plus kits (cat#: 3321-4HPW-2, Mabtech, Stockholm, Sweden), Mouse TNF-α ELISpot plus kits (cat#:3511-4HPW-2, Mabtech), Mouse IL-2 ELISpot plus kits (cat#: 3441–4HPW-2, Mabtech), and Mouse IL-4 ELISpot plus kits (cat#: 3311-4HPW-2, Mabtech) were used to detect interferon (IFN)-γ-, tumor necrosis factor (TNF)-α-, interleukin (IL)-2- and interleukin (IL)-4-positive cells. Ninety-six-well PVDF plates were rinsed twice with 200 μL of PBS and blocked with RPMI-1640 medium containing 10% fetal bovine serum for 2 h at 25 °C. Subsequently, isolated lymphocytes (2.5 × 10^5^ cells) were added to each well (two replicates) and treated (excluding unstimulated wells) with 1 μg/mL protein peptide pools (each peptide segment consists of 13 amino acids, with 11 amino acids repeated) from ZIKV EDIII (Genscript, Nanjing, China) and ZIKV NS1 (Genscript) for 24 h at 37 °C. Lymphocytes were then incubated with anti-mouse IFN-γ, anti-mouse TNF-α, anti-mouse IL-2 or anti-mouse IL-4 antibodies for 2 h at ambient temperature, followed by incubation with streptavidin-horseradish peroxidase (1:1000) for 1 h. Cells were washed with 3,5,3′,5′-tetramethylbenzidine (TMB, 100 μL) substrate solution prior to incubation for 5 min until visible spots appeared. Spots from IFN-γ-secreting cells were imaged and counted using an ImmunoSpot S6 universal spot counter (Cellular Technology Limited, Shaker Heights, OH, USA).

### 2.5. Intracellular Cytokine Staining

Isolated splenocytes were stimulated with 1 μg/mL (2 μg/mL total) protein peptide pools from ZIKV EDIII (Genscript) and ZIKV NS1 (Genscript) and terminated cytokine transport using 2 μg/mL BD GolgiPlug™ Protein Transport Inhibitor (containing brefeldin A) (cat#: 555029, BD, Franklin Lakes, NJ, USA) for 8 h at 37 °C. Cells were then stained with a mixture of PE hamster anti-mouse CD3e antibody (cat#: 553063, BD), BV510 rat anti-mouse CD4 antibody (cat#: 563106, BD), FITC rat anti-mouse CD8a antibody (cat#: 553030, BD), and Fixable Viability Stain 780 (cat#: 565388, BD) to exclude dead cells. Cells were washed twice with PBS, permeabilized with Cytofix/Cytoperm (cat#: 555028, BD), washed with Perm/Wash buffer, and stained with PE-Cy™7-conjugated rat anti-mouse IFN-γ (cat#: 557649, BD), PerCP-Cy™5.5 Rat Anti-Mouse TNF (cat#: 560659, BD), BV605 rat anti-mouse IL-2 (cat#: 563911, BD) and APC Rat Anti-Mouse IL-4 (cat#: 554436, BD). After washing with PBS and Perm/Wash buffer, cells were resuspended in PBS and analyzed by flow cytometry. At least 50,000 cells were acquired per sample. Data were analyzed using FlowJo software (V10.8.1). CD4^+^ and CD8^+^ T cells were gated based on single cells (FSC-A vs. FSC-H), live CD3^+^ T cells (CD3^+^ vs. FVS780^−^), and lymphocytes (FSC-A vs. SSC-A). Flow-based Gating Scheme in Supplementary Figure S1. Results are expressed as the percentage of cytokine-positive CD4^+^ or CD8^+^ T cells.

### 2.6. Analysis of Antigen-Specific IgG Titers Using Enzyme-Linked Immunosorbent Assays (ELISA)

ELISA was used to analyze antigen-specific antibody titers in serum. Ninety-six-well plates were coated overnight with antigen proteins from ZIKV EDIII (2 μg/mL) (cat#: RM2591, Vazyme) and ZIKV NS1 (2 μg/mL) (cat#: RM2592, Vazyme). After washing with PBST (PBS + 0.05% Tween-20), wells were blocked with PBST containing 1% bovine serum albumin for 1 h at 37 °C. Following six washes with PBST, serially diluted serum was added to each well (two replicates) and incubated for 1 h at 37 °C. Plates were washed six times with PBST, then probed with horseradish peroxidase-conjugated goat anti-mouse IgG (1:25,000) for 1 h at 37 °C. After shaking, 3,5,3′,5′-tetramethylbenzidine was used as the substrate, and absorbance was measured at 450 and 630 nm. Serum IgG titers were determined using GraphPad Prism v.9 software. Serum antibody titers were defined as the maximum dilution at which absorbance was 2.1-fold higher than that of the negative control.

### 2.7. Analysis of Serum Neutralizing Titers Using Plaque Reduction Neutralization Tests (PRNTs)

Vero cells were digested with trypsin-EDTA (0.25%) solution at room temperature, and digestion was terminated with high-glucose DMEM containing 10% FBS. All liquid was collected and centrifuged at 90× *g* for 3 min, and the supernatant was discarded. Cells were resuspended in high-glucose DMEM with 10% FBS, counted, and viability was assessed. Cells were diluted to 1 × 10^5^ cells/mL (based on viable cells) and seeded at 1 mL per well in 24-well plates, then cultured for approximately 24 h at 37 °C with 5% CO_2_. Mouse serum was serially diluted in DMEM starting at 1:5 with twofold gradients up to 1:160 and transferred to 96-well plates at 100 μL per well. ZIKV was diluted in DMEM to 250 PFU/mL, and 100 μL was added per well to the 96-well plate. After thorough mixing with serum samples, the 96-well plate was incubated at 37 °C for 90 min, with shaking every 15 min to ensure virus-serum contact. Post-incubation, 200 μL from each well was transferred to the corresponding 24-well cell plate with proper labeling. Cell plates were gently shaken to distribute the virus evenly and incubated at 37 °C for 1 h, with shaking every 20 min. Methylcellulose overlay was added at 1 mL per well, and plates were cultured for 6 days. Plates were removed, the overlay was aspirated, and 500 μL of crystal violet stain was added to each well for 40 min at room temperature. The stain was gently rinsed with water, and excess moisture was absorbed with paper towels. Plaques on the plate bottom were counted and recorded for each well. The titer is defined as twice the highest dilution at which the plaque count is half that of the virus control group.

### 2.8. Statistical Analyses

Experimental data were analyzed using GraphPad Prism v9, with figures optimized and presented using Adobe Illustrator 2023. Comparisons were performed by one-way ANOVA and Tukey’s multiple comparison tests, with significance thresholds set as follows: * *p* < 0.05; ** *p* < 0.01; *** *p* < 0.001; **** *p* < 0.0001; ns indicates no statistical significance (*p* > 0.05).

## 3. Results

### 3.1. Construction and Characterization of mRNA Vaccines

mRNA sequence design primarily consists of the 5′ cap, 5′ UTR, coding region, 3′ UTR, and polyadenylated tail. The 5′ cap is an N7-methylated guanosine that forms a 5′-ppp bond with the 5′-most nucleotide of the mRNA to create m7GpppN. By binding to translation initiation factors, it protects the mRNA from degradation by nucleases; it also facilitates the initiation of translation and prevents recognition by the innate immune system. The 5′ UTR contains ribosome recognition sequences that, in conjunction with the 3′ UTR, initiate the translation process and mediate the subcellular localization of the mRNA; the polyadenylated 3′ end primarily maintains the stability of the mRNA (Figure 1A). The bicomponent Zika mRNA vaccine utilized the EDIII domain and NS1 protein of ZIKV (FSS13025 strain) as antigens. These proteins were human codon-optimized and cloned into separate plasmids containing untranslated regions (UTRs) and a poly(A) tail (Figure 1B). mRNA-LNPs were prepared by encapsulating the two types of mRNA separately with LNPs.

The malvern particle size analyzer showed that the resulting EDIII mRNA-LNPs had an average particle size of 70.15 nm and a polydispersity index (PDI) of 0.119, and the NS1 mRNA-LNPs had an average particle Z-average hydrodynamic diameter of 79.52 nm and a PDI of 0.145 (Figure 2). The encapsulation efficiencies of the two vaccines were 83.00% and 81.48%, respectively. The two mRNA vaccines were directly transfected into human embryonic kidney (HEK) 293 T cells in 24-well plates at concentrations of 2 μg/well. Untransfected cells were used as negative controls. Target proteins were detected in the cell lysates 48 h after transfection using the Zika virus (strain Zika SPH2015) Envelope Protein (ZIKV-E) ELISA Kit (cat#: KIT40543, Sino Biological, Beijing, China) and the Zika virus (strain Zika SPH2015) ZIKV-NS1 ELISA Kit (cat#: KIT40544, Sino Biological). ELISA results showed that both mRNA vaccines successfully expressed the target proteins in vitro (E DIII: 214.52 ng/mL; NS1:199.10 ng/mL).

### 3.2. Comparison of Cellular Immune Responses Triggered by Bicomponent and Monocomponent Vaccines

To streamline the cellular immunity section and focus on the immunologically relevant doses, we chose to present the 10 µg and 20 µg groups, as the 5 µg dose yielded minimal cellular responses. BALB/c mice were administered the EDIII, NS1, or EDIII + NS1 vaccine (10 and 20 μg) or a placebo. Mice were euthanized, and splenocytes were collected from each group 35 days after primary immunization. Lymphocytes were stimulated for 24 h with peptide pools derived from the EDIII and NS1 proteins of ZIKV (FSS13025 strain). Following peptide pool stimulation, the secretion levels of four cytokines—IFN-γ, TNF-α, IL-2, and IL-4—were higher in the bicomponent vaccine groups than in the monocomponent vaccine groups. In particular, levels of IFN-γ and IL-2, which are closely associated with cellular immune responses, were significantly higher in the bicomponent vaccine groups than in the monocomponent vaccine groups.

Post-stimulation, ELISpot was used to detect the specific secretion of IFN-γ, IL-2, IL-4, and TNF-α in the EDIII and NS1 groups (Figure 3). The bicomponent vaccine induced significantly higher cellular immune responses in mice than those for the monocomponent EDIII vaccine. Levels of IFN-γ and IL-2 secretion in the bicomponent vaccine groups were significantly higher than those in the monocomponent vaccine groups. In the 10 μg dose group, the arithmetic mean titers of spot-forming units (SFU) per 2.5 × 10^5^ splenocytes in the bicomponent vaccine group targeting ZIKV EDIII and NS1 were 315.1 and 112.5, respectively, compared with 104.6 and 34.1 in the monocomponent vaccine groups—representing 3-fold and 3.3-fold increases. In the 20 μg dose group, these increases were 3.5-fold and 3.2-fold, respectively. These findings indicate that the nonstructural protein NS1 plays a critical role in enhancing cellular immunity; its combination with the EDIII vaccine provides more robust cellular immune protection, thereby offering more comprehensive host defense.

### 3.3. mRNA Vaccines Induce Th1-Biased Cellular Immunity in Mice

We evaluated the Th1/Th2 bias of the mRNA vaccine-specific T-cell immune responses. Splenocytes were harvested from mice and subjected to intracellular cytokine staining following stimulation with ZIKV EDIII and NS1 protein peptide pools to determine whether the mRNA vaccines induced Th1-biased T-cell immune responses against different mouse subsets. As shown by intracellular cytokine staining (Figure 4 and Figure 5), in CD4-positive splenocytes, the low-dose monocomponent EDIII vaccine group exhibited significantly higher levels of CD4-positive secretory IL-2 and IFN-γ cytokines than those in the phosphate-buffered saline (PBS) group, while the high-dose group showed significantly higher levels of CD4-positive secretory IL-2, TNF-α, and IFN-γ cytokines relative to levels in the control. The high- and low-dose bicomponent EDIII + NS1 vaccine groups both showed significantly higher levels of CD4-positive secretory IL-2, TNF-α, and IFN-γ cytokines than those in the PBS group. Neither vaccine group showed higher CD4-positive IL-4 cytokine levels than those in the PBS group. In CD8-positive splenocytes, the high-dose monocomponent EDIII vaccine group showed significantly higher levels of CD8-positive secretory IFN-γ than those in the PBS group. The low-dose bicomponent EDIII + NS1 vaccine group showed significantly higher levels of CD8-positive secretory IL-2 and IFN-γ than those in the PBS group, while the high-dose group showed significantly higher levels of CD8-positive secretory IL-2, TNF-α, and IFN-γ than those in the PBS. Neither vaccine group showed higher CD8-positive IL-4 cytokine levels than those in the PBS. These results indicate that both mRNA vaccines induced Th1-type cellular immunity. Notably, wide individual variation was observed within groups, particularly in bicomponent vaccine groups, which may reflect heterogeneous cellular uptake of dual-antigen LNPs. Caution is warranted in interpreting between-group differences given the small sample size (n = 5).

### 3.4. mRNA Vaccines Induce High Levels of Serum-Specific IgG Antibodies

ELISA was used to detect EDIII- and NS1-specific IgG titers in the 5, 10, and 20 μg dose groups to evaluate the humoral immune response levels induced by the mRNA vaccines in mice. The test result was total specific IgG in serum. As shown in Figure 6, both vaccines produced significantly higher antibody titers than those in the negative control group. Specifically, the EDIII vaccine produced average antibody titers of 1:7040, 1:43,520, and 1:92,160 in the 5, 10, and 20 μg groups, respectively, while the NS1 vaccine produced titers of 1:3840, 1:6400, and 1:71,680 in the corresponding dose groups. These results demonstrate that the prepared mRNA vaccines induce high levels of binding antibodies, a critical prerequisite for vaccine efficacy.

### 3.5. ZIKV EDIII Vaccine Induces High Levels of Neutralizing Antibodies

PRNT50 experimental results, as shown in Figure 7, revealed that the EDIII vaccine-immunized group exhibited the highest neutralizing antibody levels, followed by the bicomponent vaccine group. Furthermore, neutralizing antibody titers were positively correlated with the EDIII vaccine dose. Even in the bicomponent vaccine, neutralizing antibody levels were associated solely with the EDIII component. The NS1 protein, a nonstructural protein absent in mature virions, did not induce detectable neutralizing antibody titers. PRNT_50_ titers showed substantial inter-individual variation within each dose group. Between-group comparisons should be interpreted cautiously given n = 5 per group; trends are described rather than definitive conclusions where *p*-values approached but did not reach significance.

### 3.6. Immunoprotective Effects of the Bicomponent Vaccine in Mice

As shown in Figure 8, under intraperitoneal challenge with 10^5^ PFU ZIKV, the bicomponent vaccine exhibited robust protective efficacy. All bicomponent vaccine groups achieved complete protection in AG129 mice. In terms of mortality, the monocomponent EDIII vaccine provided suboptimal protection in AG129 mice, with the 20 μg dose group failing to confer complete protection. The NS1 vaccine demonstrated favorable immunoprotective effects, with complete protection (100% survival) at 20 μg and 80% survival at 10 μg in AG129 mice.

## 4. Discussion

This study successfully designed and constructed expression elements and vectors for a bicomponent mRNA vaccine and obtained high-purity template plasmids via synthetic biology techniques.

In particular, two mRNA vaccine products were successfully prepared. Evaluation of vaccine protein expression via ELISA showed that both components of the prepared vaccine products were successfully expressed in HEK 293T cells. The ELISA assessment of mouse serum binding antibodies demonstrated that the bicomponent mRNA vaccine products successfully induced specific IgG against both target proteins, a prerequisite for humoral immunity. After confirming IgG induction, PRNT50 was used to evaluate neutralizing antibody levels within the specific IgG, revealing that the structural protein EDIII elicited a moderate level of neutralizing antibodies, whereas the nonstructural protein NS1 did not induce neutralizing antibodies. Although PRNT_50_ is widely used for evaluating vaccine-induced neutralizing antibody responses, more stringent criteria such as PRNT_90_ have been employed in some Zika virus studies to better distinguish cross-reactive flavivirus antibodies. Future studies may benefit from including PRNT_90_ titers to further evaluate the breadth and specificity of the neutralizing antibody response elicited by this vaccine candidate.

Evaluations of bicomponent vaccines in different ratios showed that neutralizing antibody levels were associated solely with the EDIII structural protein dose. This confirms that non-structural proteins do not induce the production of neutralizing antibodies.

In the cellular immunity evaluation, mouse spleens were stimulated with target protein peptide pools for analyses of splenocyte secretory cytokine levels.

Flow cytometry results indicated that the mRNA vaccines primarily induced Th1-type cellular immunity. Flow cytometry results showed that the levels of IFN-γ, IL-2, and TNF-α secretion in the immunized group were significantly higher than those in the PBS group, while there was no significant change in IL-4 secretion. This indicates that the prepared mRNA vaccine primarily induces a Th1-type cellular immune response in BALB/c mice. Since the mRNA vaccine antigen is primarily produced by translation in the host cell’s ribosomes, the induction of a Th1-type cellular immune response is consistent with its technical principles. In Yan’s study [19], using C57 mice, the modified Zika mRNA vaccine also induced a Th1-biased cellular immune response. The capacity of monocomponent vaccines to induce secretory cytokine-producing splenocytes was inferior to that of the bicomponent vaccine based on analyses of cytokine diversity and expression levels, with the bicomponent vaccine inducing higher levels of CD8-positive secretory IL-2 and TNF-α cytokines. ELISpot results showed that secretory cytokine expression levels (except for IL-4 levels) in the bicomponent vaccine groups under peptide pool stimulation were significantly higher than those in monocomponent groups.

A protection against virus challenge study revealed that the bicomponent vaccine provided superior protection compared with that of monocomponent vaccines, with the monocomponent NS1 vaccine outperforming the EDIII vaccine. These findings validate the inclusion of NS1 as a vaccine antigen. The inferior protective efficacy of the monocomponent EDIII mRNA-LNP compared to the NS1 formulation likely reflects fundamental differences in their immunobiology. While EDIII is the primary target for neutralizing antibodies, it is a relatively small domain (approximately 50 amino acids) that may be susceptible to proteolytic degradation or misfolding when produced in vivo from mRNA, potentially limiting its accessibility to B cells. In contrast, NS1 is a multifaceted protein that forms stable dimers and hexamers, acting as a potent danger signal that robustly activates dendritic cells. This leads to stronger CD4^+^ and CD8^+^ T cell priming, which we hypothesize is the major driver of the enhanced viral control observed here. Future studies employing T cell depletion will be required to confirm this mechanistic hypothesis. In challenge experiments, the 10 μg bicomponent vaccine group (EDIII + NS1) achieved 100% protection in AG129 mice, significantly outperforming the monocomponent EDIII vaccine (20 μg group protection rate < 100%). Moreover, the monocomponent NS1 vaccine exhibited better protective effects than those of EDIII, confirming the role of NS1. This is consistent with the finding reported by Liu et al. [13].

Anti-NS1-associated non-neutralizing antibodies are critical to provide protection against Zika virus disease; this has been proven in previous studies with in vitro and in vivo experiments [19,23,24]. Antibody-bound NS1 can promote the clearance of NS1-expressing cells by effector cells through antibody-dependent cellular cytotoxicity (ADCC) mediated by Fc-γ receptors.

The observations that EDIII exhibits insufficient immunogenicity due to its small molecular weight, while NS1, despite enhanced protection, cannot directly neutralize the virus in early infection, emphasize the need to optimize E protein design to increase neutralizing epitope exposure and to investigate whether NS1-mediated immunoprotection involves complement inhibition or TLR4 signaling pathway regulation. Furthermore, the long-term safety of the vaccine and its cross-protective efficacy against neurotropic ZIKV strains require further validation. While the studies suggest a pivotal role for T cells, we acknowledge the limitation of not having performed passive transfer experiments using purified memory CD4^+^ or CD8^+^ T cells into naïve AG129 recipients. Such experiments would definitively establish the protective capacity of cellular immunity independent of antibodies. Given the suggestive evidence in the current study, we propose to conduct these adoptive transfer studies in future work to formally delineate the contributions of humoral versus cellular effectors. A limitation of the present study is the small group size (n = 5), which limited statistical power and may have contributed to wide individual variation. Larger confirmatory studies are warranted to validate the observed immunological and protective trends.

A major limitation of this study is that viral load testing and analysis were not performed on the challenge mice. Given that AG129 mice lack type I and type II interferon receptors, their protective mechanisms may differ from those of normal mice. Analysis of viral load would provide further insight into the vaccine’s protective mechanism. The significant role of NS1 as an antigen warrants further investigation; its combination with the structural protein E DIII produces a stronger protective effect. A critical safety concern for Zika vaccines is the potential to induce antibody-dependent enhancement (ADE) of dengue virus (DENV) infection. Our construct was designed to mitigate this risk by utilizing NS1 and the EDIII domain of the E protein. Unlike the prM protein or the EDI/II domains, which harbor the cross-reactive epitopes implicated in DENV ADE, NS1 and EDIII are largely non-cross-reactive and have not been associated with enhancing antibodies in preclinical models [24]. While this strategic antigen selection theoretically minimizes ADE risk, we acknowledge that formal in vivo ADE assessments (e.g., passive transfer followed by DENV challenge in AG129 mice or non-human primates) are warranted in future studies to definitively rule out this safety liability. It is important to note that the mRNA vaccine construct described herein was designed based on the envelope protein sequence of the FSS13025 strain, representative of the Asian lineage circulating during the recent outbreaks. Consequently, the protective efficacy demonstrated in this study pertains specifically to the Asian lineage. The potential to confer cross-protection against the genetically distinct African lineage remains to be evaluated and represents a critical next step in the development of this vaccine candidate.

The results of this study highlight important distinctions among three conceptually different outcomes of vaccination: (i) immunogenicity, (ii) neutralizing antibody induction, and (iii) protective efficacy. NS1 mRNA vaccination induced high titers of antigen-specific binding antibodies as measured by ELISA and robust Th1-biased cellular responses characterized by IFN-γ production; in this sense, NS1 is clearly immunogenic. However, NS1-specific antibodies were undetectable in PRNT_50_ assays, which is expected because NS1 is not present on the virion surface and therefore cannot be targeted by entry-blocking neutralizing antibodies. This dissociation between ELISA positivity and PRNT_50_ negativity underscores that total binding antibody titers do not predict neutralizing capacity when the antigen is a non-structural protein.

Importantly, despite the absence of detectable neutralizing antibodies, NS1 immunization conferred partial protection against a lethal ZIKV challenge (20 μg group), demonstrating that protective efficacy can be mediated by mechanisms other than viral neutralization. These mechanisms likely include Fc-dependent effector functions (ADCC, ADCP, CDC) and T-cell-mediated viral clearance, as discussed above. The failure of the 10 μg NS1 group to achieve comparable survival (>50% mortality) further suggests that a threshold level of NS1-specific immunity is required for meaningful protection.

In light of these findings, we qualify our earlier characterization of NS1: rather than describing NS1 as a “good immunogen” in the conventional sense of a neutralizing-antibody-inducing antigen, we propose that NS1 is better described as a protective antigen that engages non-neutralizing immune effector mechanisms. Its value in a bicomponent vaccine strategy lies precisely in this complementarity—EDIII provides neutralizing antibodies, while NS1 contributes Fc-effector and cellular immunity, yielding synergistic protection that neither component achieves alone.

## 5. Conclusions

In this study, we successfully developed a bicomponent Zika mRNA vaccine and conducted immunological evaluations of it. In vitro experiments successfully validated the accurate expression of antigenic proteins by the bicomponent mRNA vaccine, and we conducted a systematic evaluation of the vaccine’s humoral and cellular immune responses. In humoral immune response experiments, the bicomponent vaccine successfully induced the production of corresponding specific binding antibodies in mice, and it was confirmed that neutralizing antibodies were primarily induced by the E DIII vaccine. In cellular immunity evaluation experiments, mice immunized with the bicomponent vaccine exhibited significantly higher cytokine levels in splenocytes stimulated with antigenic peptides compared to those immunized with the single-component vaccine, indicating a higher level of cellular immunity. The NS1 protein significantly enhanced the vaccine’s cellular immune response. In the mouse viral challenge protection assay, the bivalent vaccine provided superior immune protection to AG129 mice compared to both monovalent vaccines. Meanwhile, the NS1 vaccine also demonstrated good protective efficacy, indicating that the NS1 protein holds significant value for Zika vaccine development and should be prioritized as a key direction for future Zika vaccine research.

In summary, the newly developed bicomponent mRNA vaccine, by synergistically activating humoral and cellular immune pathways, offers a novel strategy for ZIKV prevention and control; its design rationale may also serve as a reference for the development of vaccines against other flaviviruses.

## Figures and Tables

**Figure 1:**
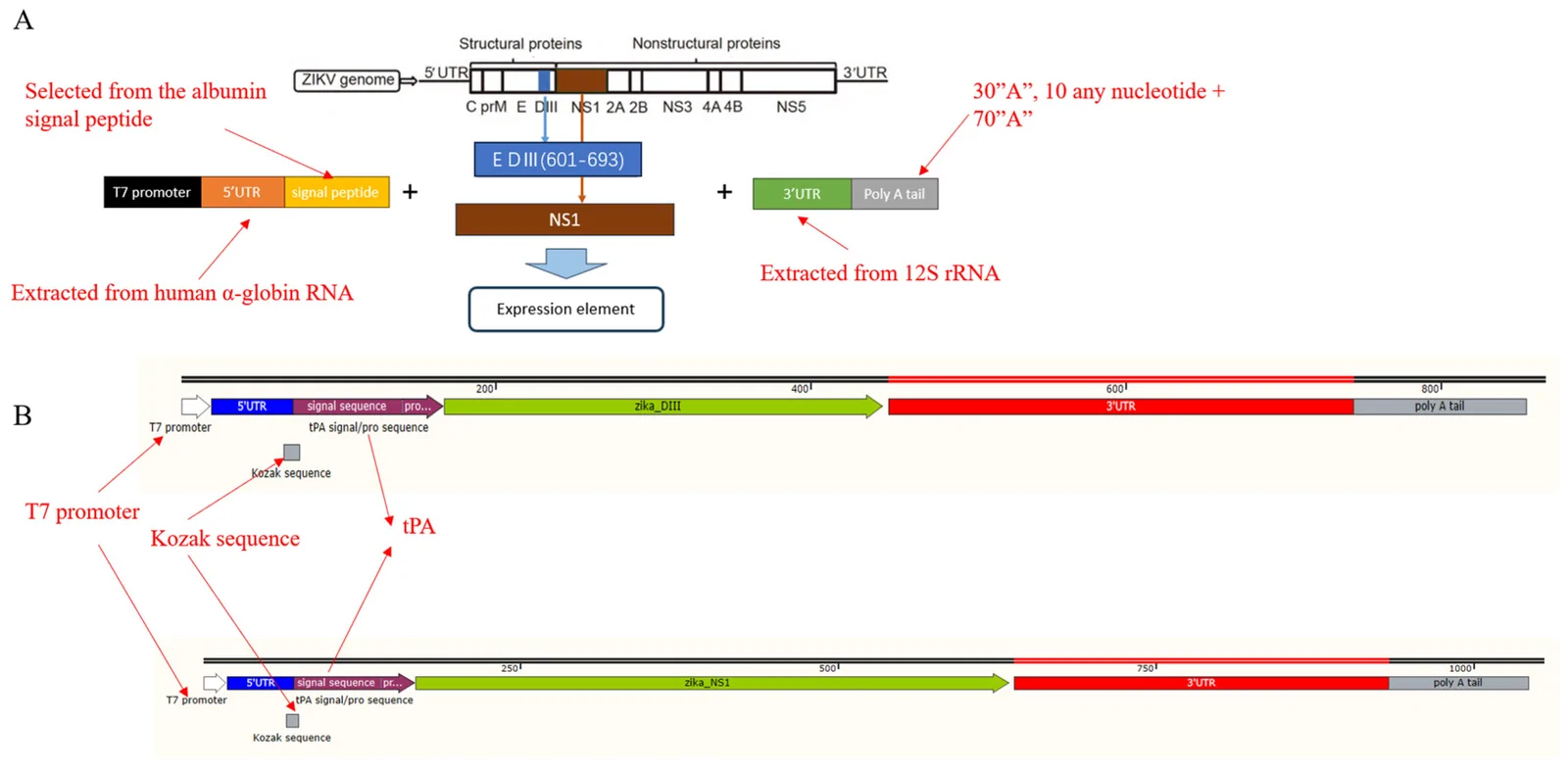
Schematic Diagram of mRNA Vaccine Expression Vector Design. (**A**) illustrates the design concept of the expression vector, and (**B**) shows a schematic diagram of the completed expression vector.

**Figure 2:**
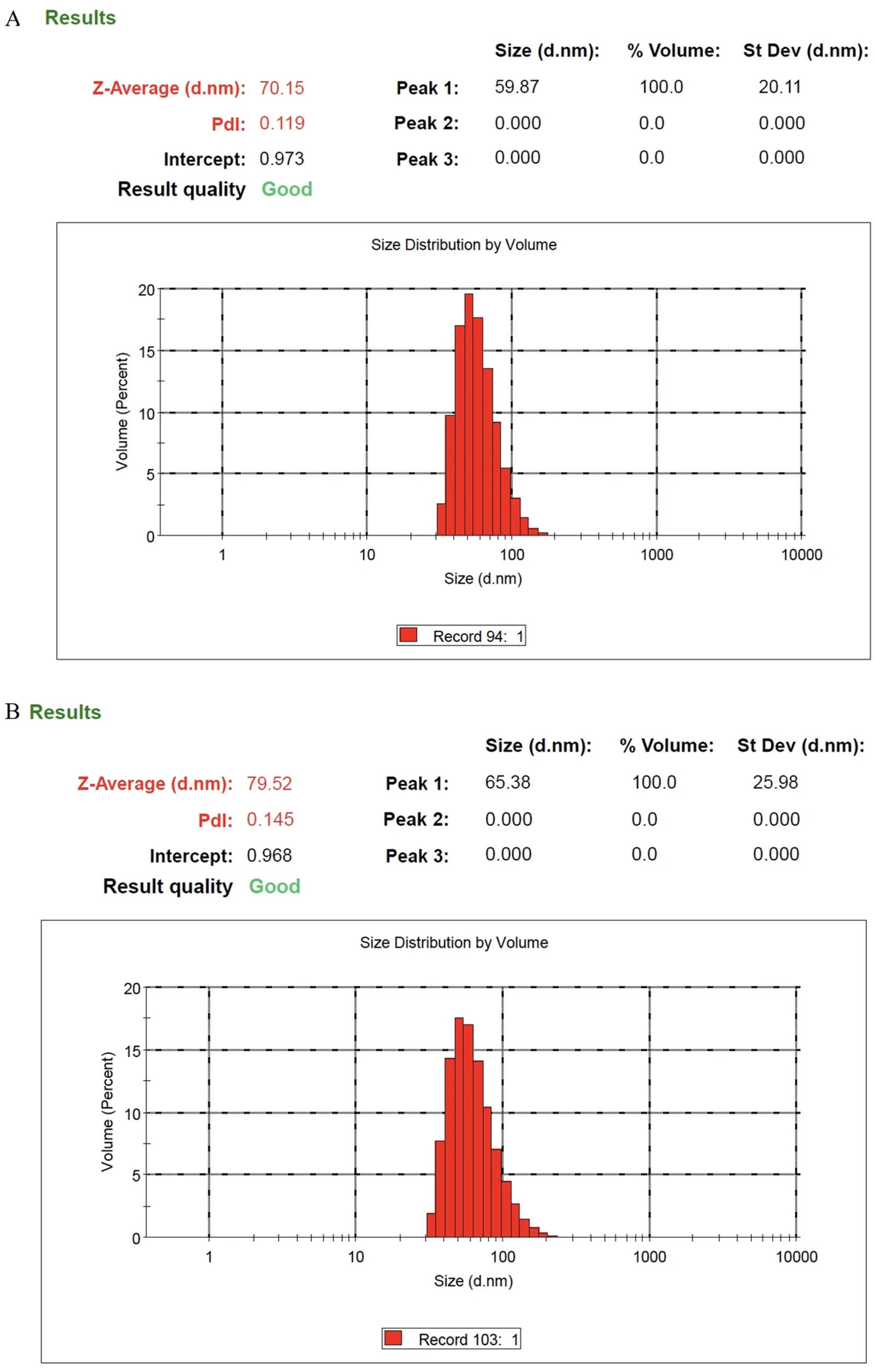
Particle Z-average hydrodynamic diameter distribution of lipid nanoparticles containing the mRNA constructs encoding EDIII (**A**) and NS1 (**B**).

**Figure 3:**
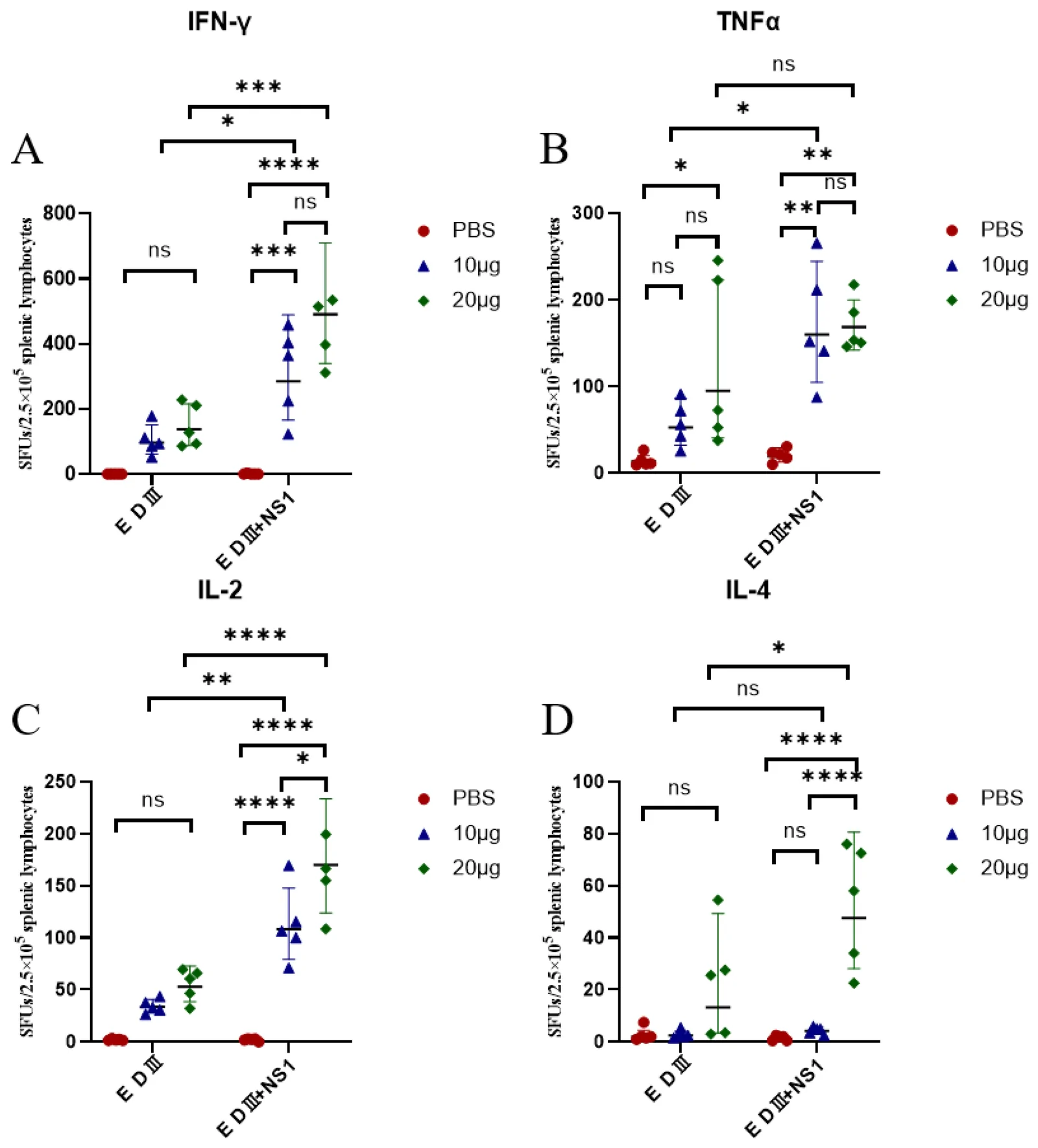
ELISpot assay results evaluating antigen-specific cellular immune responses. After stimulation with a peptide library, mouse spleen cells were analyzed using an ELISpot assay kit. (**A**) shows the results for IFN-γ; (**B**) shows the results for TNF-α; (**C**) shows the results for IL-2; and (**D**) shows the results for IL-4. Comparisons were performed by one-way ANOVA and Tukey’s multiple comparison tests; * *p* < 0.05; ** *p* < 0.01; *** *p* < 0.001; **** *p* < 0.0001; ns indicates no statistical significance (*p* > 0.05).

**Figure 4:**
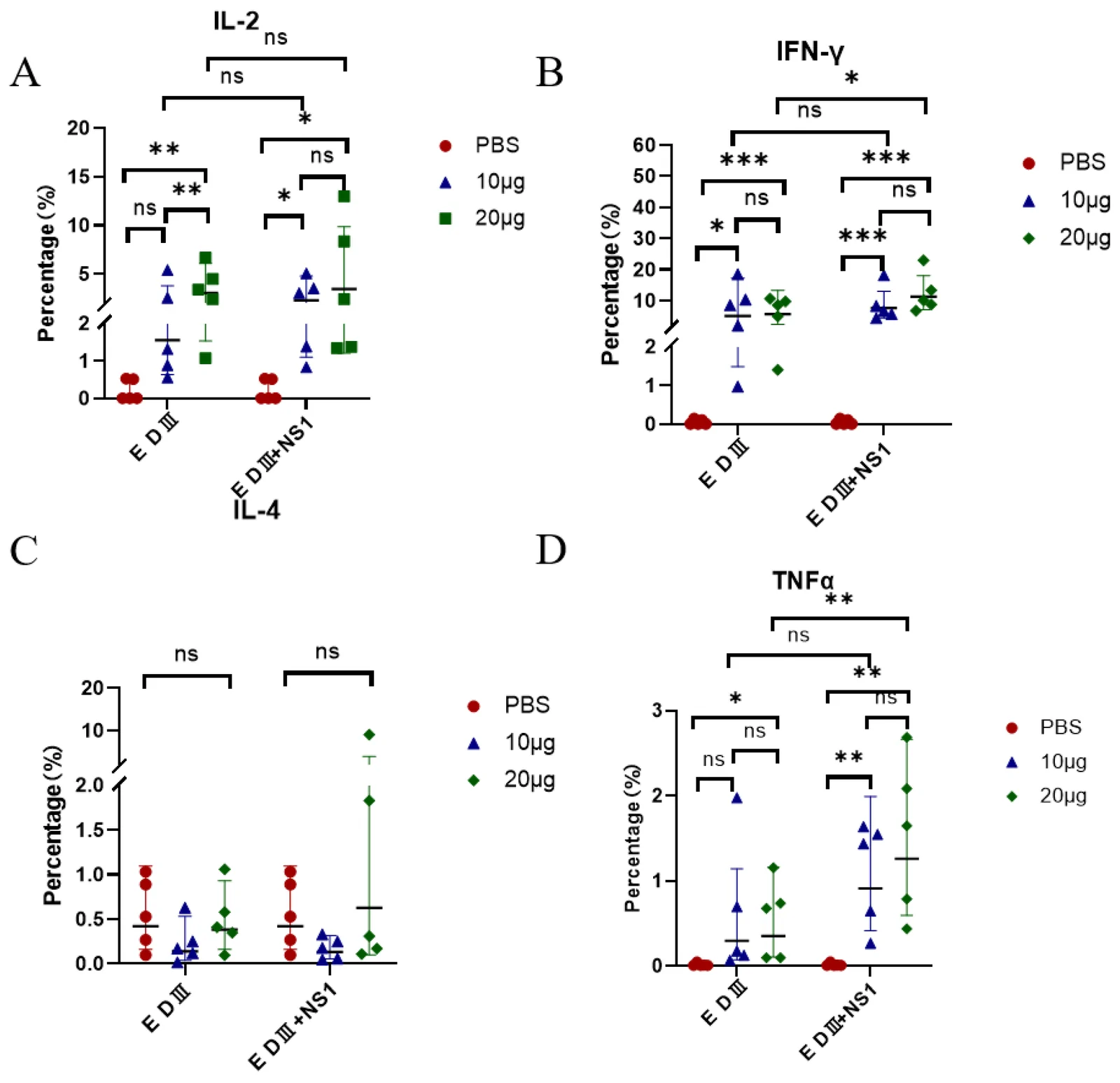
Flow cytometry assay CD4^+^ T cell results evaluating the level of cellular immunity: (**A**) IL-2 results; (**B**) IFN-γ results; (**C**) IL-4 results; and (**D**) TNF-α results. Comparisons were performed by one-way ANOVA and Tukey’s multiple comparison tests; * *p* < 0.05; ** *p* < 0.01; *** *p* < 0.001; ns indicates no statistical significance (*p* > 0.05).

**Figure 5:**
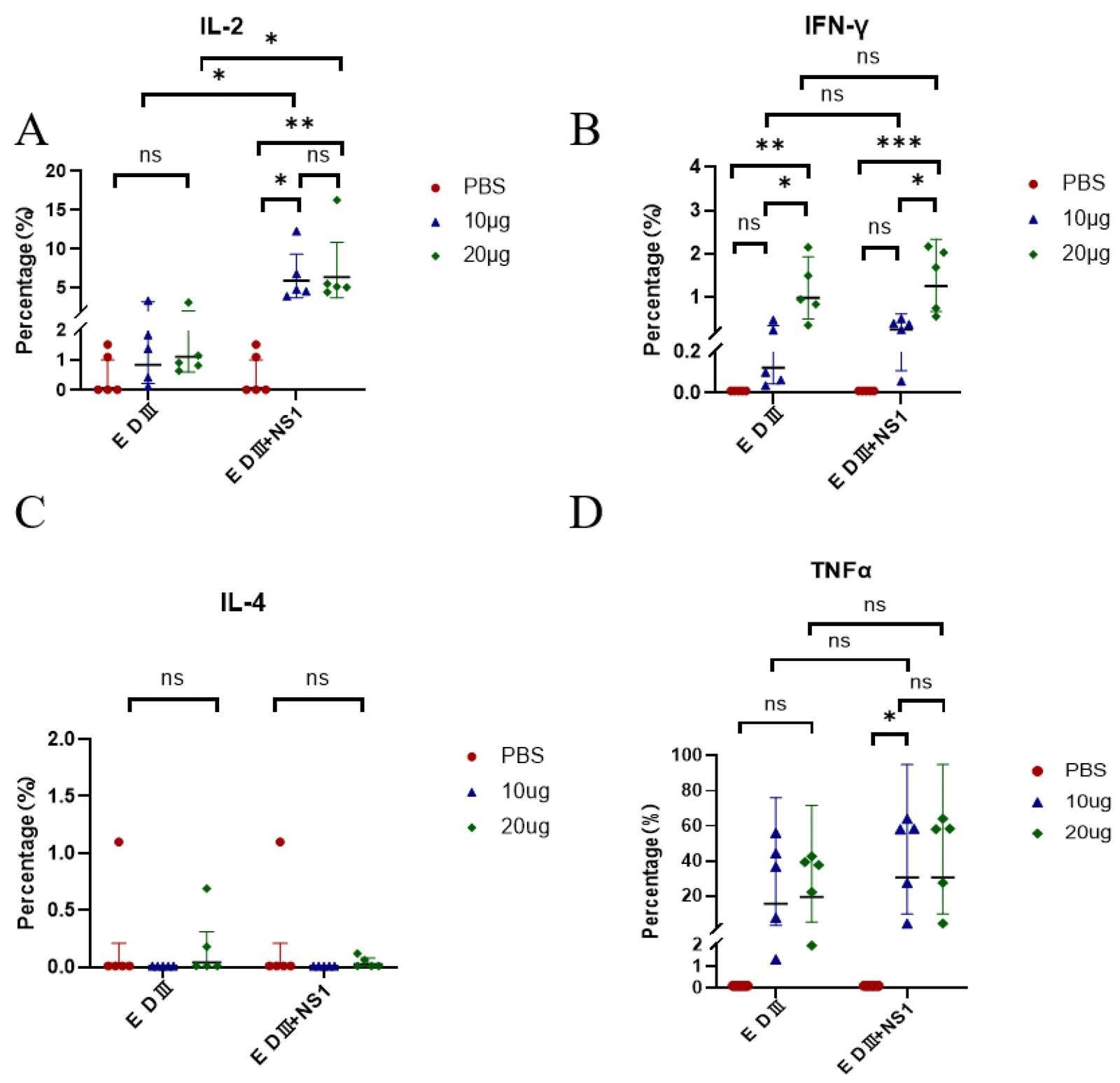
Flow cytometry assay CD8^+^ T cell results evaluating the level of cellular immunity: (**A**) IL-2 results; (**B**) IFN-γ results; (**C**) IL-4 results; and (**D**) TNF-α results. Comparisons were performed by one-way ANOVA and Tukey’s multiple comparison tests; * *p* < 0.05; ** *p* < 0.01; *** *p* < 0.001; ns indicates no statistical significance (*p* > 0.05).

**Figure 6:**
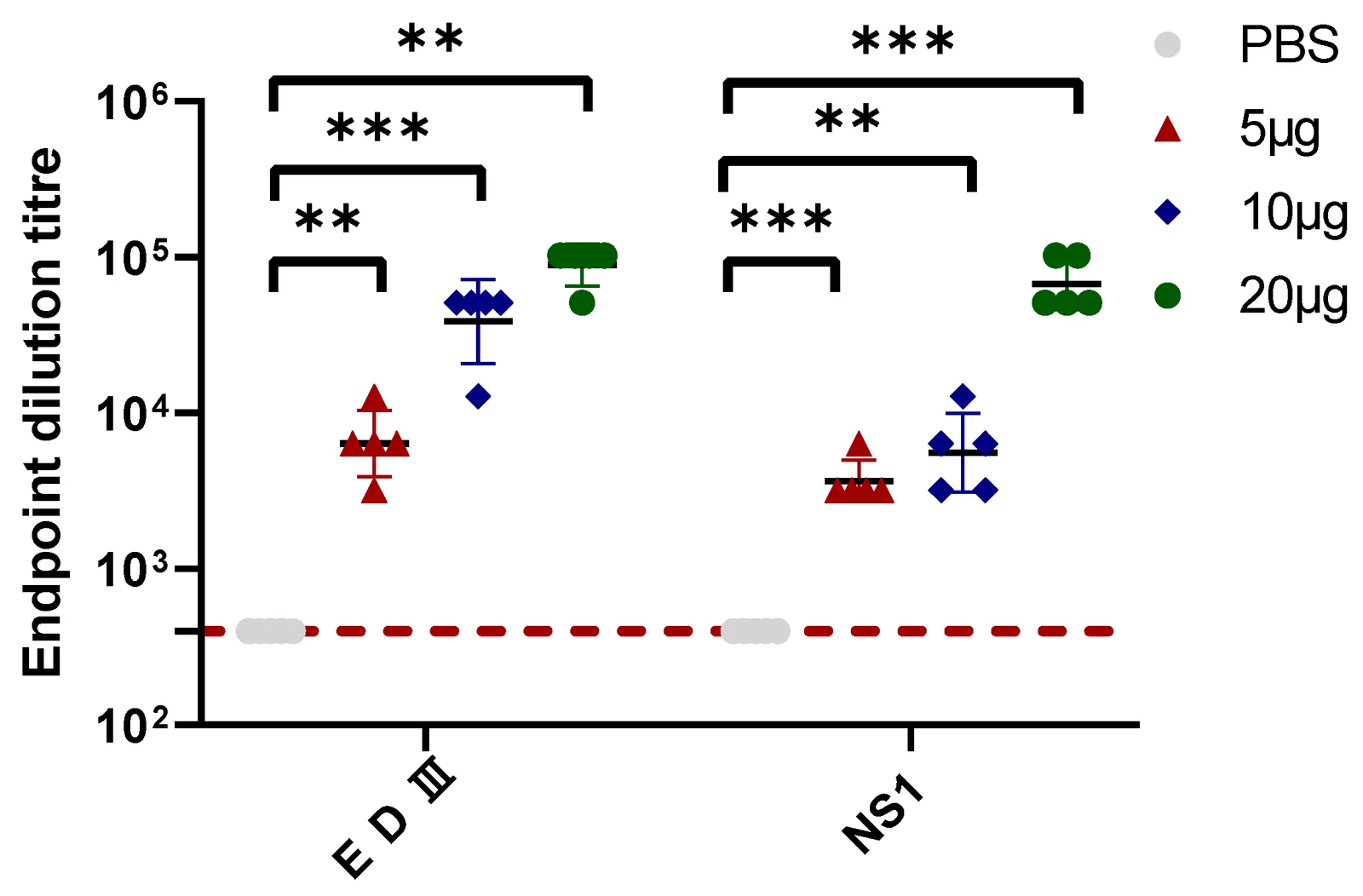
ELISA results evaluating mouse serum-specific IgG titer determination. When detecting specific antibodies in mouse serum using an ELISA, EDIII and NS1 proteins were used as coating antigens (both at a concentration of 2 μg/mL). The cutoff value was set at 2.1 times the absorbance value of the negative serum, and the endpoint titer of the sample was calculated. Comparisons were performed by one-way ANOVA and Tukey’s multiple comparison tests; ** *p* < 0.01; *** *p* < 0.001.

**Figure 7:**
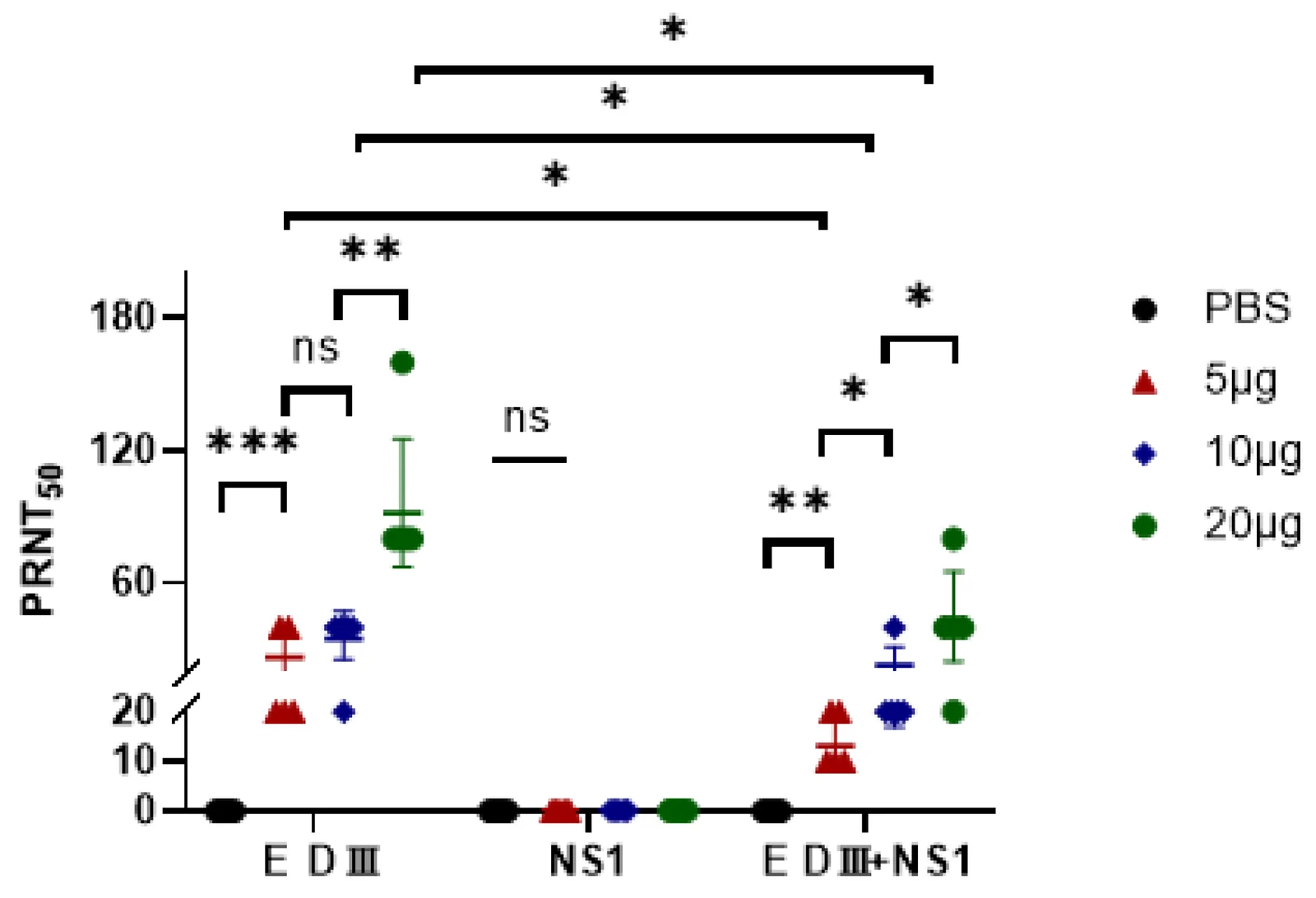
PRNT50 assay results evaluating serum neutralizing antibody titer. Represents neutralizing antibody titers in different dose groups of the three vaccines. Comparisons were performed by one-way ANOVA and Tukey’s multiple comparison tests. * *p* < 0.05; ** *p* < 0.01; *** *p* < 0.001; ns indicates no statistical significance (*p* > 0.05).

**Figure 8:**
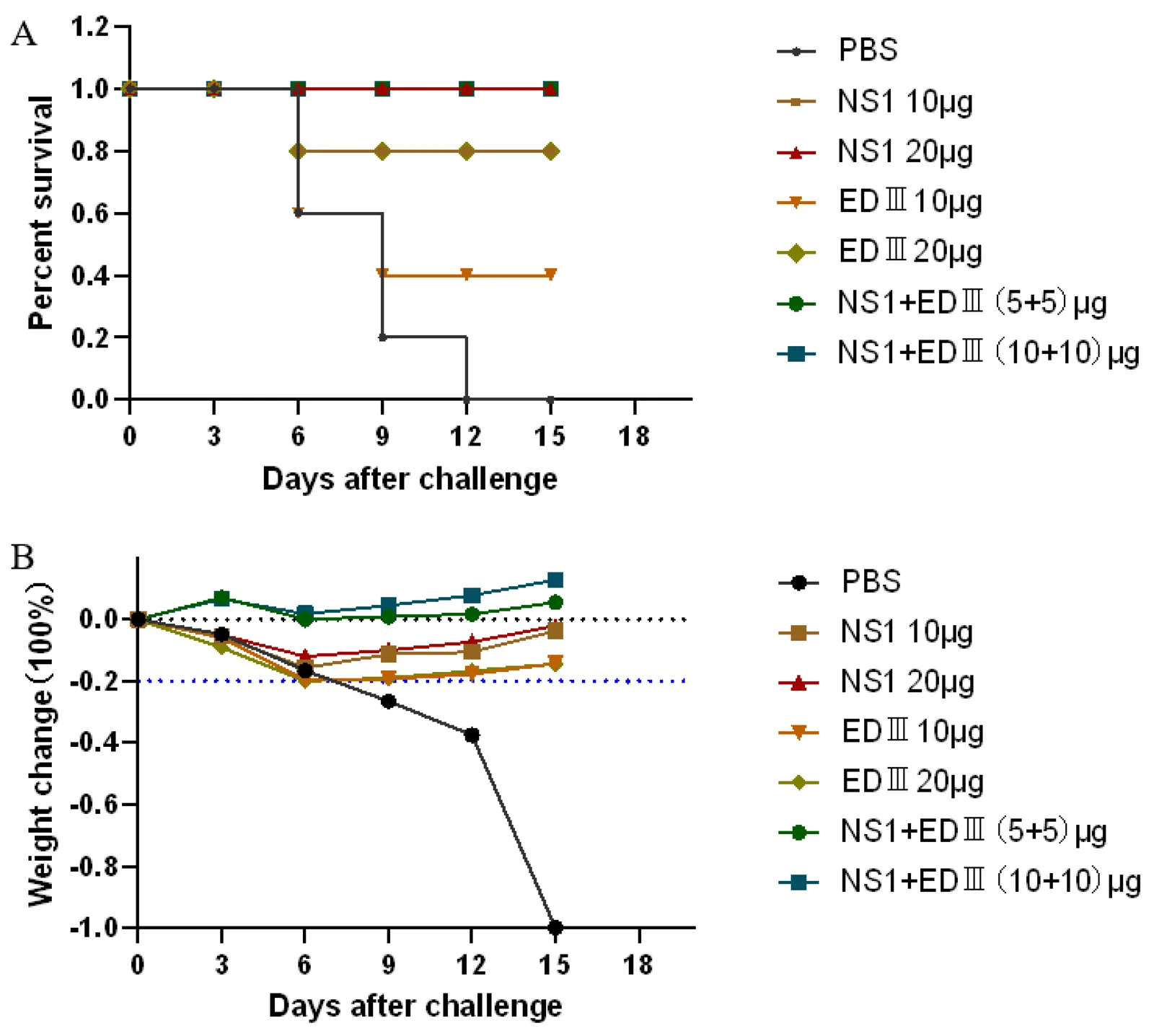
Immunoprotective effects in challenge trials. (**A**) Mouse survival rate post-challenge. (**B**) Mouse weight rate post-challenge. Comparisons were specified using Kaplan–Meier with log-rank testing for survival and repeated-measures analysis for weight trajectories.

## Data Availability

The original contributions presented in the study are included in the article. Further inquiries can be directed to the corresponding authors.

## Supplementary Materials

The following supporting information can be downloaded at https://www.mdpi.com/article/10.3390/vaccines14100897/s1; Figure S1: Flow-based Gating Scheme.

## Abbreviations

The following abbreviations are used in this manuscript:

ZIKV	Zika virus
E proteins	Envelope glycoproteins
DENV	Dengue virus
ADE	Antibody-dependent enhancement
NS	Nonstructural proteins
tPA	Tissue-type plasminogen activator
LNPs	Lipid nanoparticles
ATCC	American Type Culture Collection
NIDC	National Institutes for Drug Control
ELISA	Enzyme-linked immunosorbent assay
ELISpot	Enzyme-linked immunospot
TNF	Tumor necrosis factor
IFN	Interferon
IL	Interleukin
TMB	3,5,3′,5′-tetramethylbenzidine
UTRs	Untranslated regions
HEK	Human embryonic kidney
SFU	Spot-forming units
