# Supplementary material for: Construction and Immunological Evaluation of a Bicomponent Zika mRNA Vaccine
Source: Vaccines (Basel). 2026 Oct 13;14(10):897. doi: 10.3390/vaccines14100897 (PMC13709802; doi:10.3390/vaccines14100897)
Supplement: Supplementary file 1 [file vaccines-14-00897-s001.zip › vaccines-4522978-supplementary.pdf]

Supplementary Materials

Construction and Immunological Evaluation of a Bicomponent Zika mRNA Vaccine

Qi Su [1,2,†], Tingyi Che [1,2,†], Shuai Shang [1,2], Xinzhu Wang [1,2], Xiaoqin Ge [3], Fang Cai [3], Hongyu Wang [2], Hongshan Xu [2], Qunying Mao [2], Xiaohong Wu [2,*] and Xinyu Liu [2,*]

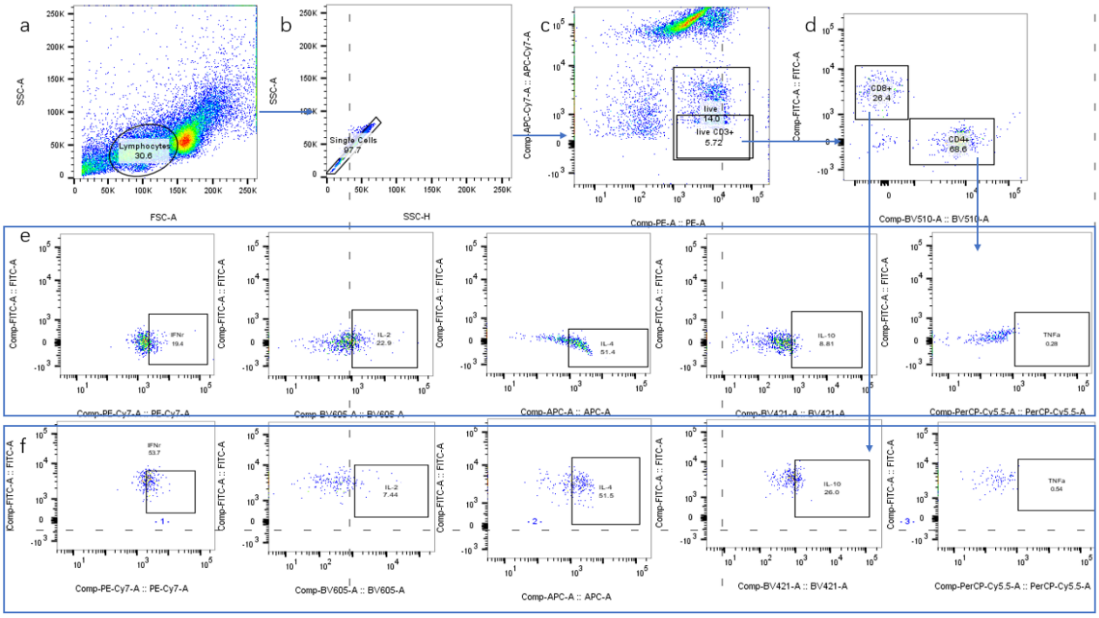

Supplementary Figure S1. Flow-based Gating Scheme

Note: Figure a shows the target cell region; Figure b shows the population of single cells; Figure c shows the population of live CD3+ cells; Figure d shows the populations of CD4+ T cells and CD8+ T cells; Figures e and f show the populations of CD8+ and CD4+ cells that are positive for the cytokines INF-γ, TNF-α, IL-2, IL-4, and IL-10, respectively.
